# Metformin as an Innate Immune Modulator: Metabolic and Epigenetic Reprogramming of Innate Immune Cells and Therapeutic Implications

**DOI:** 10.3390/cimb48060642

**Published:** 2026-06-22

**Authors:** Yunfeng Shi, Sheng Xia

**Affiliations:** Department of Immunology, School of Medicine, Jiangsu University, Zhenjiang 212013, China

**Keywords:** metformin, innate immunity, macrophage, immunometabolism, epigenetic regulation, AMP-activated protein kinase

## Abstract

Metformin, widely prescribed for type 2 diabetes mellitus (T2D), has emerged as a systemic immunomodulator with effects that extend far beyond glycemic control. Recent advances in immunometabolism reveal that metformin modulates innate immune responses through coordinated cellular metabolic reprogramming and epigenetic modification, which collectively modulate the functional phenotype of innate immune cells. This narrative review summarizes current evidence regarding the immunomodulatory effects of metformin on the innate immune system, with a focus on immunometabolism and epigenetic regulation. It explores how metformin modulates innate immunity by altering cellular energy sensing, mitochondrial function, and nutrient utilization. Such metabolic changes and alterations further reshape chromatin structure and architecture, as well as transcriptional profiles and programs. Through the regulation of glycolysis, fatty acid oxidation, and histone modification landscapes, metformin regulates the phenotypes of innate immune cells, which can be pro-inflammatory, tolerogenic, or homeostatic. This conceptual framework presents a new understanding of metformin. As well as acting as an anti-inflammatory agent, it may regulate immune memory.

## 1. Introduction

The immune system is now recognized as a metabolically driven network in which cellular function is tightly coupled to bioenergetic status. Over the past decade, research in immunometabolism has yielded important findings about metabolic pathways in immune cells. Such pathways are not just passive mechanisms supporting immune cell survival but actively influence cell differentiation and effector functions. Glycolysis, oxidative phosphorylation (OXPHOS), and lipid metabolism are also key factors influencing whether immune cells adopt an inflammatory, regulatory, or memory-like phenotype. Within this framework, the concept of trained immunity has revolutionized traditional views of immunological memory [1]. Innate immune cells, once considered incapable of forming immunological memory, can undergo long-term functional reprogramming following transient external stimulation [2]. This process is mediated by sustained metabolic rewiring and epigenetic remodeling, enabling these cells to mount altered responses upon secondary challenge with homologous or heterologous stimuli [1]. While trained immunity enhances host defense in certain contexts, maladaptive training immunity driven by metabolic stress contributes to chronic inflammation, cardiometabolic disease, and cancer [3,4,5].

Metformin, a first-line therapy for T2D, occupies a unique position at the intersection of metabolism and immunity. Classically recognized for improving insulin sensitivity via the inhibition of mitochondrial complex I and activation of AMP-activated protein kinase (AMPK), metformin is now appreciated to exert broad immunomodulatory effects. Metformin exerts complex immunomodulatory effects beyond simple anti-inflammatory activity. Its ultimate outcomes vary depending on the disease type, basal metabolic status of the organism, local tissue microenvironment, targeted immune cell subsets, and drug concentrations. Under different circumstances, metformin can suppress excessive inflammation, induce immune tolerance, and activate anti-tumor and anti-infectious immunity; meanwhile, it may also lead to immunosuppression. It modulates macrophage polarization, dendritic cell (DC) activation, neutrophil function, and lymphocyte differentiation, primarily through metabolic mechanisms. Notably, many of these pathways overlap with those that govern the induction and maintenance of trained immunity.

The innate immune system mounts a rapid, non-specific response against external pathogens and internal damage signals. Its components encompass physical and chemical barriers, including skin, mucous membranes, and antimicrobial peptides, as well as immune cells such as macrophages, neutrophils, DC, and natural killer (NK) cells. These cells recognize pathogen-associated molecular patterns and damage-associated molecular patterns via pattern recognition receptors, thereby initiating inflammatory responses to combat infection and promote tissue repair. Beyond pathogen defense, innate immunity primes adaptive immune responses through the activation of antigen-presenting cells such as DC, thereby establishing a functional bridge between innate and adaptive immunity [6,7].

Emerging evidence demonstrates that metformin influences innate immunity by reprogramming cellular metabolism and immune cell function. Its primary mechanisms involve AMPK activation and modulation of the mammalian target of rapamycin (mTOR). Metformin promotes anti-inflammatory M2 macrophage polarization while suppressing pro-inflammatory M1 responses. It enhances NK cells’ cytotoxicity through improved metabolic flexibility and regulates DC maturation and antigen presentation, thereby modulating T-cell priming.

The gut microbiota acts as a crucial metabolic mediator linking host physiological functions and immune regulation [8]. Accumulating evidence has demonstrated that metformin remodels the composition and function of gut microbiota: It increases the abundance of Bifidobacterium, Akkermansia, and short-chain-fatty-acid-producing microbes while reducing the proportion of pro-inflammatory bacteria [9]. Microbial metabolites such as short-chain fatty acids, secondary bile acids, and tryptophan metabolites regulate the metabolic patterns, epigenetic status, and inflammatory levels of innate immune cells via activating GPR41/43, the aryl hydrocarbon receptor (AhR), and the farnesoid X receptor (FXR) [10].

Innate immune dysfunction is implicated in chronic inflammatory diseases, autoimmune disorders (e.g., rheumatoid arthritis, inflammatory bowel disease), and cancer. Metformin’s immunomodulatory properties render it a promising therapeutic candidate for these conditions [11,12,13]. Nevertheless, the precise molecular mechanisms underlying its effects on innate immune cells remain incompletely understood. This article reviews research into the interaction between metformin and the innate immune system, including its effects on innate immune cells and its role in regulating immune metabolic pathways and the epigenetic landscape.

## 2. Method

Literature retrieval was performed using PubMed and Web of Science. These two databases comprehensively cover basic experimental and clinical studies, ensuring the completeness and representativeness of literature sources. The search formula was formulated as follows: (Metformin [MeSH] OR metformin OR dimethylbiguanidine OR glucophage) AND (immunity OR immune OR innate immunity OR adaptive immunity OR immune response OR inflammation OR inflammatory OR pro-inflammatory OR anti-inflammatory OR cytokine OR chemokine OR interleukin OR TNF OR interferon OR TLR OR NLRP3 OR macrophage OR monocyte OR neutrophil OR eosinophil OR mast cell OR dendritic cell OR DC OR NK cell OR natural killer cell OR ILC OR innate lymphoid cell OR glycolysis OR fatty acid oxidation OR FAO OR epigenetics OR epigenetic). The retrieval deadline was 10 April 2026. Preclinical studies included in vitro cell experiments, in vivo animal experiments, and research on molecular mechanisms. Clinical evidence consisted of human-based studies such as clinical trials, cohort studies, and case–control studies. Literature screening was conducted in two steps: Irrelevant and duplicate articles were initially excluded by titles and abstracts, followed by a full-text review to verify content relevance. In addition, studies with obvious methodological flaws and low credibility were eliminated based on experimental design, data integrity, and standardization of research implementation to finalize the literature inclusion.

## 3. Pharmacokinetic Profiles, Intracellular Accumulation, and Tissue Distribution of Metformin

The pharmacokinetic properties, intracellular accumulation, and tissue distribution patterns of metformin serve as critical links between its effective in vivo exposure and immunomodulatory functions. As a drug primarily taken up via organic cation transporters of the solute carrier (SLC) family (e.g., OCT1), its tissue distribution is closely correlated with the expression levels of these transporters [14]. Following oral administration, metformin accumulates markedly in the gastrointestinal tract, liver, and kidneys. The peak plasma concentration reaches approximately 20–30 μM at a dose of 1 g, and the intestine has been identified as one of its major sites of action [15,16]. Notably, clozapine inhibits OCT1-mediated hepatic uptake in a dose-dependent manner, which reduces intrahepatic drug distribution and attenuates the hypoglycemic effect of metformin. This indicates that the transporter function exerts a decisive impact on tissue drug exposure [14].

Cellular uptake is a prerequisite for metformin to exert biological effects. Transporters such as OCT1 mediate the entry of metformin into hepatocytes, fetal hepatocytes, and HEK293-OCT1 cells. Silencing OCT1 substantially impairs the cellular uptake of metformin and the subsequent glucose consumption effect [14,17]. Once inside cells, metformin activates the AMPK pathway by regulating cellular energy status, while some of its immunological effects are mediated through AMPK-independent mechanisms, thereby modulating the functions of various immune cells [18,19]. For instance, in natural killer (NK) cells, metformin enhances cytotoxic activity via the p38 MAPK-dependent JAK/STAT and AKT/mTOR pathways in an AMPK-independent manner [18]. In CD8^+^ T cells, it downregulates MYC to reduce the competitive tryptophan uptake by tumor cells and restore T cell function [20]. In dendritic cells, metformin induces a tolerogenic phenotype; decreases the expression of MHC-II, co-stimulatory molecules, and pro-inflammatory cytokines (TNF-α and IFN-γ); upregulates IL-10 and PD-L1; and facilitates the differentiation of regulatory T cells [21]. In macrophages, it directly enhances antibacterial activity via metabolic reprogramming and AMPK activation, and it also exhibits anti-inflammatory potential [13,22].

In terms of tissue distribution, metformin accumulates dramatically in tissues with high transporter expression, such as the liver and intestine, and its concentration in tumor tissues is higher than that in adipose tissue [16,23]. Such distribution characteristics enable metformin to effectively act on the local immune microenvironment: it promotes the infiltration of NK cells and T cells into tumor tissues [18], inhibits myeloid-derived suppressor cells (MDSCs), and elevates the CD8^+^/Treg ratio and the expression of T cell inflammation-related genes [24]. Furthermore, in models of bone defects and neuroinflammation, local delivery strategies (e.g., hydrogel scaffolds and nanocarriers) can improve drug retention in target tissues, synergistically facilitating osteogenesis, angiogenesis, and immunomodulation [25,26].

In conclusion, the immunomodulatory effects of metformin are highly dependent on its effective accumulation in specific tissues and cells. Transporter expression, administration strategies, and microenvironmental conditions collectively determine its pharmacological efficacy. These findings provide a theoretical basis for optimizing the application of metformin in cancer immunotherapy, inflammatory diseases, and other related fields [27,28].

## 4. Metformin Modulates the Functional Fate of Key Innate Immune Cells

Innate immune cells mainly include macrophages, DC, neutrophils, NK cells, and innate lymphoid cells (ILCs). Metformin participates in regulating cell function by inducing metabolic reprogramming and epigenetic alterations.

### 4.1. Macrophage Polarization: Metformin-Mediated Metabolic Regulation of M1/M2 Fate Decisions

Macrophages are highly plastic innate immune cells that can be polarized by lipopolysaccharides (LPS)/interferon-gamma (IFN-γ) into pro-inflammatory, antimicrobial M1 or by interleukin-4 (IL-4)/IL-13 into anti-inflammatory, tissue-reparative M2 phenotypes [29,30]. The balance between these states is critical for immune homeostasis, whereas dysregulation contributes to disease pathogenesis [31,32,33,34]. Macrophage polarization is intimately coupled to cellular metabolism: M1 cells predominantly utilize glycolysis, whereas M2 cells rely on fatty acid oxidation and OXPHOS.

In vitro cellular studies have demonstrated that metformin modulates inflammatory responses in macrophages via activating AMPK. Early work demonstrated metformin-induced AMPK activation in hepatocytes, thereby altering cellular metabolism and inflammatory signaling [35]. Metformin attenuates monocyte-to-macrophage differentiation, downregulates pro-inflammatory cytokine production, attenuates oxidative stress, and promotes M1-to-M2 phenotypic transition. In human vascular smooth muscle cells, macrophages, and endothelial cells, metformin suppresses IL-1-induced IL-6 and IL-8 production and inhibits nuclear factor-κB (NF-κB) signaling [36]. In models of saturated fatty acid (FA)-induced low-grade inflammation, metformin attenuates tumor necrosis factor-α (TNF-α) and IL-6 expression and secretion in RAW264.7 macrophages via AMPK activation, demonstrating its anti-inflammation capacity [37]. Co-culturing of umbilical cord mesenchymal stem cells (UC-MSCs) with M1 and M2 macrophages revealed the increased expression of osteogenic genes (*Alp*, *Runx2*, *Ocn*, and *Col1a1*) in UC-MSCs [38]. These results suggest that metformin-primed macrophages may regulate UC-MSC function and possess therapeutic implications for inflammatory diseases and cancer. Notably, in the tumor microenvironment, metformin can antagonize cancer-induced M2 polarization, upregulate M1-associated cytokines (IL-2, TNF-ɑ), and downregulate M2-associated cytokines (IL-8, IL-10, and transforming growth factor-β (TGF-β)). This effect is partially mediated via the AMPK-NF-κB signaling pathway, thereby remodeling the immunosuppressive tumor niche [39].

Metformin functions through AMPK-independent pathways as well. Reactive oxygen species (ROS) scavenging represents another AMPK-independent mechanism underlying the biological activities of metformin. Algire et al. reported that metformin reduces ROS production, thereby protecting cells against DNA damage and mutagenesis [40]. Nguyen et al. further demonstrated that metformin suppresses lithocholic acid (LCA)-induced ROS generation, which in turn blocks the NF-κB signaling pathway. This pathway is essential for the upregulation of interleukin (IL)-8 in human colorectal cancer (CRC) HCT116 cells [41]. Beyond its direct cellular effects, metformin also modulates macrophage function indirectly via the gut microbiota. Metformin enriches short-chain-fatty acid-producing bacteria [42]. The resultant short-chain fatty acids activate GPR43 on the macrophage’s surface and inhibit histone deacetylase (HDAC), thereby promoting macrophage polarization toward the anti-inflammatory M2 phenotype and suppressing NOD-like receptor family pyrin domain-containing 3 (NLRP3) inflammasome activation [43].

As an intrinsic cellular factor linking metabolic pathways and signaling cascades, the NAD^+^/NADH ratio serves as a core hub governing macrophage activation [44]. During CD40-mediated antitumor immunity, glutamine-to-lactate conversion drives the reprogramming of tumor-associated macrophages (TAMs) via precise tuning of the NAD^+^/NADH ratio; ablation of key metabolic enzymes disrupts this metabolic circuit and abrogates subsequent antitumor efficacy [45]. While no existing literature has directly validated that metformin modulates macrophage activation by altering the NAD^+^/NADH ratio, the NAD^+^/NADH axis remains a highly promising therapeutic target for metformin-directed regulation of macrophage antitumor polarization.

Animal studies have also demonstrated that metformin modulates macrophage polarization by regulating multiple critical signaling pathways. In a bronchopulmonary dysplasia model, metformin attenuates lung inflammation in neonatal mice through the downregulation of Sonic Hedgehog (Shh) signaling, concomitant reduction in pro-inflammatory cytokines, and enhanced pulmonary vascular development [46]. In a rat sciatic nerve injury model, metformin-driven M2 polarization was abrogated by AMPK inhibition, implicating the AMPK/peroxisome proliferator-activated receptor γ coactivator-1α(PGC-1α)/peroxisome proliferator-activated receptor γ (PPAR-γ) axis as critical for metformin-mediated anti-inflammatory and neuroprotection effects [47,48,49,50,51,52]. Furthermore, in wound healing and tissue repair models, metformin enhances M2 polarization through NLRP3 inflammasome inhibition, thereby accelerating angiogenesis and tissue regeneration [53]. In peripheral nerve injury models, metformin induces M2 macrophage polarization via the AMPK/PGC-1α/PPAR-γ pathway, promoting functional recovery and remyelination [47]. Similarly, in osteogenic contexts, metformin upregulates anti-inflammatory markers (e.g., cluster of differentiation 206 (CD206), arginase-1(Arg-1)) while suppressing pro-inflammatory gene expression in macrophages, thereby fostering an immunoregulatory microenvironment conducive to tissue homeostasis [54]. In Ang-II-mediated atherosclerosis mouse models, metformin significantly attenuates phorbol 12-myristate 13-acetate (PMA)-induced monocyte-to-macrophage differentiation and reduces IL-1β, TNF-α, and monocyte chemoattractant protein-1 (MCP-1, CCL2) production via the AMPK-signal transducer and activator of transcription 3 (STAT3) signaling, concomitantly ameliorating atherosclerosis lesion severity [55]. Subsequent studies have established that metformin-induced AMPK activation inhibits c-Jun N-terminal kinase 1 (JNK1) and STAT3 phosphorylation, thereby reducing pro-inflammatory mediator expression and lipid accumulation [55,56]. In models of non-alcoholic fatty liver disease (NAFLD), metformin attenuates hepatomegaly, reduces micronucleus formation, and ameliorates colonic mucosal injury. Mechanistically, these effects are partially mediated through modulation of macrophage polarization via the Toll-like receptor 4 (TLR4)/myeloid differentiation primary response 88 (MyD88)/NF-κB/mitogen-activated protein kinase (MAPK) signaling axis, alterations in gut microbiota composition, and enhanced production of gut-derived short-chain fatty acids (SCFAs) [57,58,59,60]. All of these molecular mechanisms are exclusively supported by preclinical data, and their authenticity and applicability in human macrophages remain unclarified.

Literature analyses indicate the obvious context-dependency of metformin’s immunomodulatory properties. Although most preclinical models demonstrate an anti-inflammatory (M2-skewing) effect in chronic metabolic diseases (e.g., obesity, atherosclerosis), contrasting findings have emerged in acute infection and cancer settings. In certain tumor microenvironments, excessive AMPK activation by metformin has been reported to impair macrophage phagocytosis or promote an immunosuppressive phenotype, contingent upon nutrient availability (e.g., glucose deprivation) [61,62]. Furthermore, the discrepancy between in vitro findings (frequently employing supraphysiological concentrations > 1 mM) and in vivo relevance (plasma concentrations ~10–40 μM) remains a major confounding factor. Thus, the pro- versus anti-inflammatory outcomes of metformin are not binary but are dictated by specific metabolic pressures (hypoxia versus nutrient excess), disease stage, and local drug concentrations [63,64]. By contrast, clinical evidence remains limited, largely indirect, and complex. Nevertheless, accumulating data suggest that metformin prevents excessive macrophage activation and attenuates deleterious inflammation in diverse pathological contexts, including autoimmune diseases [65], cardiovascular diseases [66], and metabolic syndromes [67]. Observational studies in patients with T2D consistently demonstrate that long-term metformin treatment reduces circulating inflammatory markers (e.g., IL-6, intercellular adhesion molecule-1 (ICAM-1), and TNF-α), correlating with improved insulin sensitivity [68]. However, a systematic review and meta-analysis of randomized controlled trials reported that metformin significantly reduces C-reactive protein (CRP) levels without concomitant changes in IL-6 or TNF-α [69]. Notably, direct interventional trials specifically assessing tissue-resident macrophage polarization in non-diabetic populations remain scarce, and observed anti-inflammatory effects may be secondary to improved metabolic control. These findings underscore the substantial translational gap between mechanistic insights from animal models and clinical efficacy in humans.

**Figure 1:**
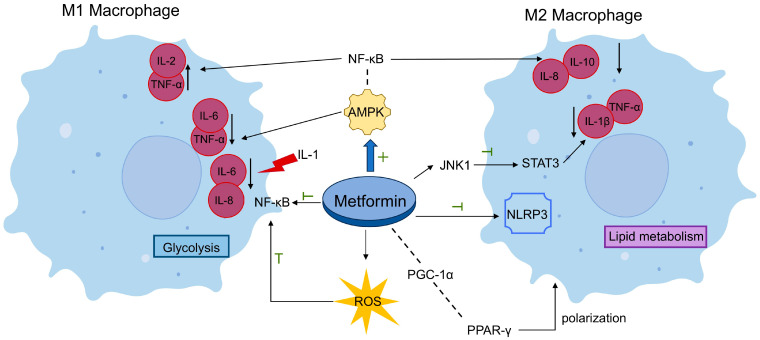
Effects of metformin on macrophages. Metformin activates AMPK, suppresses mitochondrial complex I, and modulates NF-κB, JNK, STAT3, Sirt1, and NLRP3 signaling. It represses glycolysis and pro-inflammatory cytokine (TNF-α, IL-6, and IL-1β) release in M1 macrophages while enhancing fatty acid oxidation and JAK-STAT activation in M2 macrophages. This shifts macrophage polarization from M1 to M2, mediating anti-inflammatory and metabolic regulation. “T” represents inhibition or blockade, and “+” indicates promotion or activation.

Collectively, evidence for metformin regulating macrophage polarization mainly originates from in vitro cellular experiments and animal models, and some findings have been indirectly validated in observational studies of diabetic populations. To date, no clinical intervention trials focusing on macrophage phenotypes and functions have been conducted. In addition, the drug concentrations used in in vitro experiments differ substantially from physiological concentrations in humans, and conclusions from animal studies cannot be directly extrapolated to human effects. Large-sample clinical studies are still required to further verify the relevant mechanisms and clinical application value of metformin (Figure 1).

### 4.2. Dendritic Cells: Metformin Reprograms DC Immunometabolism to Balance Immune Activation and Tolerance

DCs are professional antigen-presenting cells that occupy a pivotal position at the interface of innate and adaptive immunity, coordinating the initiation of adaptive immune responses with the maintenance of immune tolerance. DCs exist along a dynamic functional spectrum, ranging from immature DCs to mature DCs. Immature DCs reside at barrier sites, including skin, mucous membranes, and lymphoid tissues, where they enable the rapid detection of pathogens and the release of endogenous damage signals. These cells capture, process, and present antigens while expressing low levels of major histocompatibility complex (MHC) and costimulatory molecules, thereby promoting T-cell tolerance or regulatory T-cell (Treg) induction [70,71]. Upon encountering danger signals such as pathogen-associated molecular patterns (PAMPs), including LPS, DCs undergo metabolic and transcriptional reprogramming that upregulates MHC-II expression, costimulatory molecules (CD80 and CD86), and chemokine receptors (e.g., c-c chemokine receptor type 7 (CCR7) [72,73] while enhancing pro-inflammatory cytokine production (IL-12, TNF-α, and IFN-γ). These cells subsequently load antigenic peptides into MHC-II binding pockets, present them to naïve T cells, and promote effector T-cell priming through coordinated costimulatory and cytokine signals, thereby driving T-cell differentiation toward type 1 T helper cell (Th1)/Th17 lineages [70,71,74]. Thus, DC functional states reflect the integration of environmental cues and intrinsic metabolic programs that determine whether immune activation or tolerance predominates [21,73].

The maturation and activation of dendritic cells are highly dependent on surrounding immune and metabolic signals. Accordingly, the regulatory effects of metformin on these cells vary markedly under different disease conditions. In vitro studies utilizing LPS-stimulated DCs have demonstrated that metformin reduced surface expression of MHC-II, costimulatory molecules (CD80, CD86, and CD40), and pro-inflammatory cytokines and their receptors (e.g., TNF-α, IFN-γ, and CCR7), concomitantly enhancing anti-inflammatory signals such as IL-10, without compromising phagocytic activity [75]. These metformin-conditioned DCs exhibit upregulated immunomodulatory molecule expression (e.g., inducible T-cell co-stimulator ligand (ICOSL), programmed death-ligand 1 (PD-L1)), and an enhanced capacity to promote Treg differentiation. Conversely, through the AMPK-independent inhibition of mitochondrial complex I, metformin can enhance DC activation and maturation under specific conditions [76,77].

Beyond autoimmune contexts, metformin’s effects on DCs extend to diverse immunological processes. For example, studies suggest that metformin modulates DC survival and subset-specific activation, inhibiting plasmacytoid DC activation, which relies on both glycolytic and oxidative metabolism, while exerting variable effects on conventional DC populations. This functional complexity indicates that metformin’s impact on DC function is context-dependent, potentially enhancing antitumor immunity in certain models while promoting tolerance in inflammatory or autoimmune conditions [77,78]. In addition, metformin promotes the production of short-chain fatty acids and indole derivatives via gut microbiota [79]. These metabolites activate the aryl hydrocarbon receptor (AhR) and inhibit nuclear factor-κB (NF-κB), thereby driving dendritic cells to differentiate into an immune-tolerant phenotype [80]. Short-chain fatty acids upregulate the expression of programmed death-ligand 1 (PD-L1) and inducible co-stimulatory molecule ligands (ICOSLs) on dendritic cells, reduce the secretion of pro-inflammatory cytokines, and ultimately induce the generation of regulatory T cells [81].

**Figure 2:**
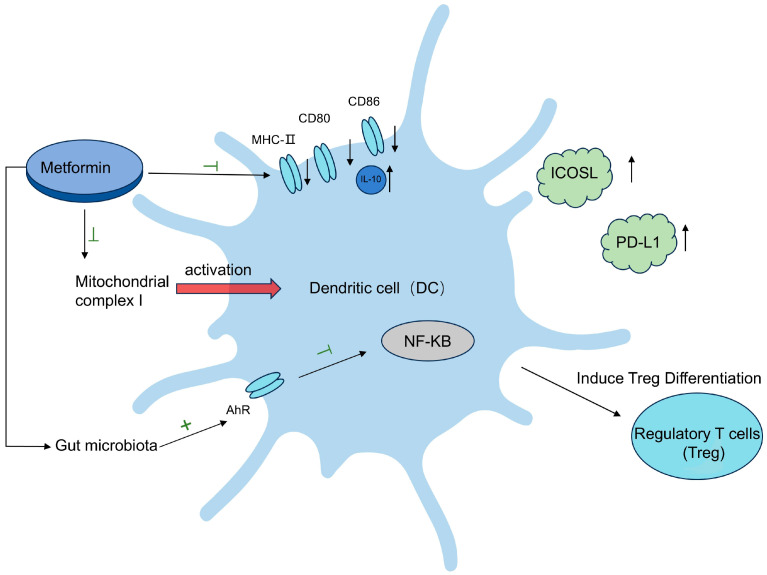
Effects of metformin on dendritic cells. Metformin inhibits mitochondrial complex I in dendritic cells and modulates expression of MHC-II and CD80/CD86. It elevates ICOSL and PD-L1. These changes remodel dendritic cell activation and maturation, boost Treg differentiation, and enhance immune tolerance. “T” represents inhibition or blockade, and “+” indicates promotion or activation.

Collectively, these findings indicate that metformin intersects with key immunometabolic circuits in DC to modulate the balance between immune activation and tolerance, positioning it as a metabolic modulator of DC fate with implications for autoimmune diseases, cancer immunotherapy, and immune homeostasis. All current evidence regarding the regulatory effects of metformin on dendritic cell function is derived exclusively from in vitro cellular experiments and animal studies, and no relevant human studies have been reported to date. The existing findings are limited by the simplistic conditions of in vitro assays, and extensive follow-up investigations are still required to accumulate data for further clinical translation (Figure 2).

### 4.3. Neutrophils: Metformin Regulates Functional Plasticity Beyond NETosis Through Metabolic Intervention

Neutrophils are traditionally characterized as short-lived, frontline effectors of innate immunity. Recruited from circulation to sites of infection, injury, or inflammation, these cells eliminate pathogens through phagocytosis, degranulation, ROS generation, and lysozyme secretion [82,83], most notably through the release of neutrophil extracellular traps (NETs). NETs are network-like structures composed of nuclear DNA, histones, and antimicrobial proteins that entrap and neutralize pathogens, thereby limiting infection dissemination [84]. Neutrophils possess strong metabolic and functional plasticity and can alter their biological behaviors in response to local inflammatory patterns and microenvironmental characteristics. Accordingly, metformin exerts distinctly different regulatory effects on neutrophils under various pathological conditions.

Beyond their fundamental antimicrobial activity, neutrophils secrete cytokines and chemokines, interact with diverse immune cells, and modulate adaptive immune responses. They are extensively involved in inflammation resolution, tissue repair, and the pathological progression of multiple diseases [85]. Their functional status is largely governed by extrinsic microenvironmental signals. Excessive release, impaired clearance of NETs, or dysregulated cell apoptosis will exacerbate inflammatory injury, and further participate in the onset and progression of acute respiratory distress syndrome (ARDS), autoimmune diseases, and malignancies [84,86,87,88].

Beyond their classical inflammatory functions, neutrophils undergo extensive metabolic reprogramming in response to environmental stresses. Under metabolic pressures, including hypoxia, nutrient deprivation, or systemic metabolic dysfunction, neutrophils intricately modulate their metabolic pathways to support specialized functions. For example, shifts between glycolytic and oxidative metabolism influence ROS generation, degranulation capacity, and migratory behavior, thereby shaping inflammatory outcomes [89]. These metabolic adaptations provide the substrate for neutrophil functional reprogramming, linking cellular metabolism to effector heterogeneity beyond the canonical neutrophil extracellular trap formation (NETosis) paradigm.

Animal studies have revealed that metformin targets and modulates neutrophil function via metabolic regulation. In a transgenic zebrafish model of acute inflammation, metformin attenuates neutrophil recruitment to inflamed tissues and dampens inflammatory cytokine expression (e.g., TNF-α, IL-1β, IL-6, and C-X-C motif chemokine ligand 8 (CXCL8)). These effects are mediated through the indirect suppression of neutrophil chemotaxis to inflammatory sites [90]. Metformin also attenuates neutrophil infiltration into myocardial tissues, thereby reducing myeloperoxidase (MPO) activity and cardiac remodeling in rat myocardial infarction models [91]. Furthermore, metformin alleviates LPS-induced acute lung injury by diminishing MPO activity and inflammatory cell infiltration, underscoring its anti-inflammatory properties. These findings highlight metformin’s potential for modulating excessive neutrophil recruitment and associated inflammatory pathologies. In ARDS, metformin enhances macrophage-mediated clearance of NETs and apoptotic neutrophils [92]. Metformin reduces NETosis and alleviates NET-mediated suppression of osteogenesis, suggesting therapeutic potential in inflammatory and tissue repair contexts [93]. In Lewis lung carcinoma (LLC) mouse models, metformin significantly attenuates tumor growth and reduces pro-inflammatory and malignancy-associated molecule expressions through modulation of cancer cell metabolism and inhibition of HMGB1-induced NETosis; additionally, it modulates obesity-driven cancer aggressiveness in high-fat-diet (HFD)-fed mice [94]. In obesity-associated pancreatic cancer, metformin counteracts NETosis pro-tumor effects by reducing PD-L1^+^ neutrophil recruitment, decreasing NETosis to 60% of control levels, promoting immune cell infiltration, and enhancing antitumor immunity [95]. In addition, metformin ameliorates pathological aberrant responses of neutrophils via remodeling the gut microbiota [96]. It lowers circulating levels of microbiota-derived lipopolysaccharide (LPS) and pro-inflammatory metabolites and attenuates neutrophil chemotaxis, oxidative stress, and NETosis [97].

Clinical studies indicate that metformin reduces the abundance of tumor-associated neutrophils (TANs) and NETs, elevates the infiltration of tumor-infiltrating lymphocytes (TILs), and suppresses tumor invasiveness [98]. Both clinical and experimental studies demonstrate that metformin reduces NETosis in endothelial cells through protein kinase C (PKC)-mediated inhibition of NADPH oxidase activation, independently of its glucose-lowering effects. This subsequently diminishes the release of NET components, including elastase, protease-3, histones, and double-stranded DNA [99]. Beyond attenuating NETosis, metformin indirectly modulates neutrophil oxidative burst and inflammatory cytokine production through AMPK activation and suppression of excessive ROS generation, thereby promoting inflammation resolution and limiting host tissue damage.

**Figure 3:**
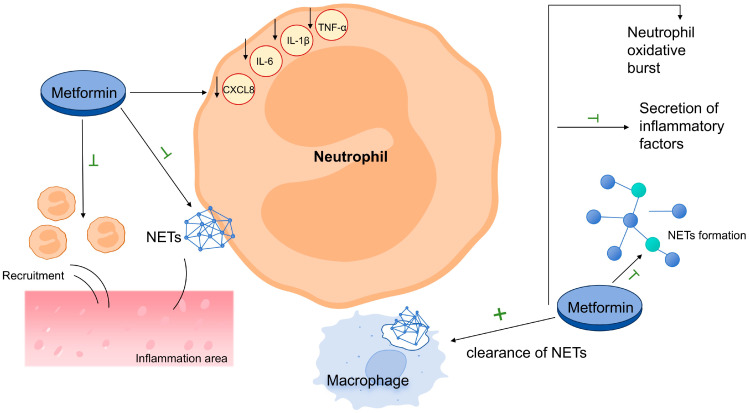
Effects of metformin on neutrophils. Metformin inhibits neutrophil pro-inflammatory activities and NET release, promotes NETs clearance, and mitigates inflammation-associated tissue damage. “T” represents inhibition or blockade, and “+” indicates promotion or activation.

Beyond these established effects, metformin’s impact on neutrophil metabolism may reshape neutrophil signaling and interactions with other immune cell subsets, potentially altering cytokine profiles and immunoregulatory functions. Beyond these established effects, metformin’s impact on neutrophil metabolism may reshape neutrophil signaling and interactions with other immune cell subsets, potentially altering cytokine profiles and immunoregulatory functions. Although detailed mechanisms remain under investigation, these findings collectively indicate that metformin functions as a modulator of neutrophil plasticity by integrating metabolic control with functional outcomes, thereby expanding its immunomodulatory role from NETosis suppression to broader regulation of neutrophil effector programs. Evidence regarding the regulation of neutrophil functional plasticity by metformin is mainly derived from animal experiments, supplemented by a small number of human observational studies. To date, no targeted clinical intervention trials have been conducted for verification. Given the interspecies differences and distinct microenvironments observed in animal studies, further in-depth validation is required for clinical translation (Figure 3).

### 4.4. NK Cells and ILCs: Metformin Modulates Cytotoxicity and Immune Homeostasis Through Metabolic Reprogramming

The innate lymphoid cell (ILC) family encompasses multiple subsets, including NK cells and other ILCs, which are classified into three subsets based on cytokine profiles: ILC1 (IFN-γ, TNF-α), ILC2 (IL-5, IL-13, IL-4), and ILC3 (IL-17, IL-22). These cells contribute to immune homeostasis and pathophysiological responses through the production of diverse effector molecules and cytokines [100]. ILCs are enriched at barrier surfaces and mucosal boundaries, constituting a heterogeneous family of effector lymphocytes that mirror T cell functionality without requiring antigen-specific recognition. Among these, NK cells function as cytotoxic sentinels that rapidly recognize and eliminate virally infected or transformed cells, including tumor cells, through perforin, granzyme-mediated cytotoxicity, antibody-dependent cellular cytotoxicity (ADCC), and cytokine secretion (e.g., IFN-γ, TNF-α), thereby executing surveillance and immune regulation [101,102]. In contrast, non-cytotoxic ILC subsets (ILC1, ILC2, and ILC3) contribute to tissue homeostasis, barrier immunity, and type-specific inflammatory responses. NK cell and ILC functional plasticity is orchestrated by distinct metabolic programs, with glycolysis and oxidative phosphorylation serving as central regulators of effector differentiation, cytokine production, and survival, thereby directly coupling cellular metabolism to immune adaptability. For instance, glycolytic metabolism supports robust NK cell cytotoxicity and IFN-γ production, whereas ILC2 and ILC3 subsets utilize a balance of glycolysis and lipid metabolism adapted to their specialized roles in type-2 and type-3 immunity, respectively. Disruption of these metabolic pathways can impair effector potency, migratory capacity, and immunoregulatory interactions within inflamed tissues [103,104].

Animal studies demonstrate that metformin modulates NK cells and ILC function by targeting cellular metabolism and energy-sensing pathways. Metformin enhances splenic NK cell cytotoxicity and alters functional phenotypes, specifically upregulating natural killer cell p46-related protein (NKp46^+^)CD3^−^CD49b^+^ and FasL^+^CD3^−^CD49b^+^ subsets while elevating cytokine production (e.g., IFN-γ) and reducing IL-10^+^ NK cell frequencies [105], thereby promoting antitumor and antiviral immunity. These alterations are associated with the modulation of miRNA regulatory networks (e.g., upregulation of miRNA-155 and miRNA-150) and downregulation of miRNA-146a, and they occur independently of IDO inhibition [105,106,107,108].

In melanoma models, metformin suppresses tumor growth by attenuating immune evasion through downregulation of PD-L1 expression in both cancer and tumor-infiltrating immune cells, including NK cells and T cells. Mechanistically, this involves reduced AKT-mediated β-catenin S552 phosphorylation and transactivation, alongside PD-1 destabilization through enhanced binding to E3 ubiquitin ligases and subsequent polyubiquitylation [109]. Furthermore, metformin increases ILC2 proportions through AMPKα1 activation, thereby promoting an immunosuppressive microenvironment that attenuates inflammation. This metabolic reprogramming improves functional outcomes in mouse models of traumatic brain injury (TBI). Although AMPK represents a primary molecular target, metformin may additionally operate through AMPK-independent pathways (e.g., mTOR/pSTAT1 in NK cells) [110]. Notably, HFD-fed mice exhibit impaired glucose tolerance and elevated circulating ILC1 and ILC3 caspase-3 expression. Metformin reduces caspase-3 activation in ILC1s through mechanisms associated with high-density lipoprotein cholesterol (HDL-c) modulation. These findings collectively indicate that metformin-induced metabolic reprogramming of ILC subsets modulates their immune functions and dampens the inflammatory response [111]. In allergic inflammation models, metformin attenuates ILC2-driven type-2 cytokine production (e.g., IL-5 and IL-13) and airway hyperreactivity, likely through AMPK-independent modulation of glycolysis and fatty acid utilization. This results in diminished ILC2 expansion and effector cytokine secretion [112].

In clinical trials involving head and neck squamous cell carcinoma (HNSCC) patients, metformin combined with cisplatin-based chemoradiotherapy (CRT) improves survival and expands activated peripheral NK cells (CD56^dim^CD16^+^). Additionally, metformin enhances NK cells’ tumor infiltration and elevates IFN-γ and perforin expression while reducing CXCL1 levels, effects mediated through mTOR and pSTAT1 pathways rather than AMPK [19].

**Figure 4:**
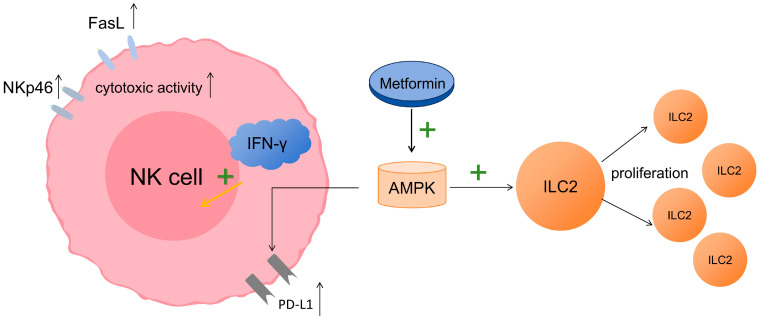
Effects of metformin on natural killer cells. Metformin enhances the cytotoxic activity of NK cells (by upregulating NKp46, FasL, and IFN-γ) and inhibits PD-L1 expression; simultaneously, by activating AMPK, it promotes the expansion of ILC2s, thereby exerting a dual effect of enhancing anti-tumor immunity and regulating inflammatory homeostasis. “+” indicates promotion or activation.

Collectively, these findings indicate that metformin reprograms metabolic pathways in NK cells and ILC subsets, thereby exerting nuanced effects on their functional dynamics. By modulating energy utilization and signaling pathways, including AMPK and mTOR, metformin enhances beneficial effector functions while preventing excessive or pathological activation. This positions metformin as a metabolic regulator of innate lymphoid effector responses with implications for oncology, infectious diseases, and inflammatory conditions. Relevant evidence regarding the regulation of NK cells and ILCs by metformin is primarily derived from animal model experiments, with only a small number of conclusions supported by clinical intervention trials. Although the overall reliability of the evidence is relatively high, the available clinical samples remain limited, and the underlying mechanisms need further validation (Figure 4).

Metformin exerts multifaceted immunomodulatory effects on four major innate immune cell lineages, encompassing both shared core mechanisms and cell-type-specific regulatory patterns. These effects collectively constitute a sophisticated regulatory network integrating metformin with innate immune cell function. Across macrophages, DC, neutrophils, NK cells, and ILCs, metformin consistently targets cellular metabolic and energy-sensing pathways. Specifically, metformin activates AMPK signaling, suppresses excessive glycolysis, inhibits mitochondrial complex I, and downregulates pro-inflammatory signaling cascades, including NF-κB, mTOR, and STAT pathways. These universal actions limit aberrant inflammatory cytokine secretion, oxidative stress, and pathological immune overactivation, thereby maintaining immune homeostasis.

Conversely, metformin mediates distinct functional reprogramming in a cell-specific manner. In macrophages, metformin drives phenotypic switching from pro-inflammatory M1 toward anti-inflammatory/tissue-repairing M2 polarization. In dendritic cells, metformin skews maturation toward a tolerogenic phenotype, thereby balancing immune activation and tolerance. In neutrophils, metformin restrains excessive recruitment, oxidative burst, and pathological NET formation while facilitating inflammation resolution. In NK cells and ILCs, metformin fine-tunes cytotoxicity, cytokine secretion, and subset composition, thereby enhancing antitumor/antiviral surveillance or alleviating type 2 inflammation. Collectively, these context-dependent, cell-specific effects enable metformin to integrate metabolic control with innate immune cell fate decisions. Table 1 summarizes the effects of metformin on different immune cell types.

## 5. Metabolic and Epigenetic Mechanisms Underlying Metformin-Mediated Innate Immune Regulation

Immunometabolism investigates the intricate interplay between the immune system and host metabolism. During immune defense, surveillance, and homeostatic maintenance, immune cells undergo substantial metabolic reprogramming. Conversely, the organismal metabolic state profoundly influences immune cell activation, proliferation, differentiation, and effector functions.

Epigenetics encompasses heritable changes in gene expression that occur without alterations to the underlying DNA sequence. Its regulatory mechanisms are multifaceted. DNA methylation, a prevalent epigenetic modification, typically occurs at cytosine residues and is frequently associated with gene silencing in eukaryotes. During tumorigenesis, promoter hypermethylation of specific tumor suppressor genes can inhibit normal gene expression, thereby promoting malignant transformations. Histone modifications, including methylation, acetylation, ubiquitination, SUMOylation, phosphorylation, lactylation, crotonylation, succinylation, butyrylation, and propionylation of histone amino acid residues, modulate chromatin structure and function, consequently altering gene accessibility.

In innate immune cells, metabolic processes and epigenetic regulation are essential for effector function execution.

### 5.1. Glycolysis: A Core Metabolic Pathway in Metformin-Mediated Attenuation of Innate Inflammation

Glycolysis, a fundamental cellular metabolic pathway, catabolizes glucose to pyruvate within the cytoplasm, generating ATP and NADH through sequential enzymatic reactions. As a cornerstone of cellular bioenergetics, glycolysis is essential for biosynthesis, proliferation, redox homeostasis maintenance, and immune cell functional regulation [113,114].

Macrophage polarization exhibits distinct metabolic dependencies, and M1 polarization relies predominantly on glycolysis, whereas M2 polarization is coupled to OXPHOS. M1 macrophages display elevated glycolytic activity accompanied by two metabolic breaks in the tricarboxylic acid (TCA) cycle, resulting in the accumulation of citrate, succinate, and lactate. Conversely, M2 macrophages maintain an intact TCA cycle and depend on OXPHOS for sustained functionality. Hypoxia-inducible factor 1α (HIF-1α) drives M1 macrophage polarization through the upregulation of glycolytic enzymes (e.g., glucose-6-phosphatase (G6PC), phosphoenolpyruvate carboxykinase 1 (PCK1)), glucose transporter 1 (GLUT1), and inflammatory mediator gene expression [115]. HIF-1α-overexpressing macrophages exhibit diminished mitochondrial oxygen consumption rates (OCRs) and enhanced extracellular acidification rates (ECARs), concomitant with elevated glycolytic intermediates and pentose phosphate pathway (PPP) metabolites [116].

Quiescent DCs depend on OXPHOS for homeostatic maintenance. However, upon PAMP engagement of Toll-like receptors (TLRs), DCs undergo metabolic reprogramming from OXPHOS toward glycolysis [117]. This metabolic shift supports immune functionality through energy provision and regulation of key glycolytic enzymes, including hexokinase, phosphofructokinase (PFK), and lactate dehydrogenase A (LDHA) [117,118,119]. This contributes to DC maturation via PI3K/AKT signaling while suppressing AMPK activity. Inflammatory neutrophils depend on glycolysis for energy generation and phagocytic activity. Glucose-6-phosphate (G6P) inhibition attenuates neutrophil phagocytosis [120]. Human neutrophils fail to generate NETs in glucose-deprived conditions, and the glycolytic inhibitor 2-deoxyglucose (2-DG) completely abrogates PMA-induced NETosis [121]. Quiescent mature NK cells primarily utilize OXPHOS to meet baseline energy requirements. Upon activation, NK cells upregulate both glycolysis and OXPHOS to satisfy elevated energy demands and support effector molecule synthesis [122].

Metformin primarily regulates glucose metabolism through the inhibition of mitochondrial complex I with the electron transport chain, thereby reducing circulating glucose and ATP production while increasing the AMP/ATP ratio in hepatocytes. This activates the energy sensor AMPK [123]. Metformin also suppresses hepatic gluconeogenesis through AMPK activation. However, hepatic gluconeogenic inhibition is not exclusively AMPK-dependent [123]. Metformin suppresses key gluconeogenic enzymes, including glucose-6-phosphatase (G6PC), whereas phosphoenolpyruvate carboxykinase 1 (PCK1) expression remains unaltered in AMPK- and liver kinase B1 (LKB1)-deficient hepatocytes. Nevertheless, conflicting reports indicate PCK1 inhibition via metformin [124,125]. Notably, glucose production suppression is enhanced in these deficient hepatocytes, suggesting dose-dependent ATP-mediated inhibition potentially involving activating transcription factor 3 (*ATF3*) [123,126]. Collectively, these findings demonstrate LKB1- and AMPK-independent gluconeogenic suppression via metformin.

In macrophages, metformin attenuates M1 polarization and inflammatory response through glycolytic suppression [127]. In neutrophils, metformin diminishes inflammatory activity, phagocytic capacity, and NETosis via attenuated glycolysis [128]. In NK cells, metformin modulates energy metabolism and cellular functionality through metabolic pathway regulation [129]. In DCs, metformin potentially influences antigen presentation and immune functionality through glycolytic modulation [21]. These metabolic regulatory mechanisms may constrain immune system hyperactivation, offering therapeutic potential in inflammatory and autoimmune conditions.

### 5.2. Fatty Acid (FA) Metabolism: Metformin Shapes Innate Immune Cell Function via FAO/FAS Modulation

Fatty acid metabolism, encompassing both catabolism (β-oxidation) and anabolism (de novo lipogenesis), is essential for energy production and cellular homeostasis. During β-oxidation, fatty acids are degraded to generate acetyl-CoA, which subsequently enters the tricarboxylic acid (TCA) cycle for ATP production. This mitochondrial process comprises three stages: activation, transport, and oxidation. De novo fatty acid synthesis occurs in the cytoplasm, where acetyl-CoA undergoes sequential enzymatic reactions to form FAs subsequently utilized for triglyceride and phospholipid synthesis [130,131].

Macrophage polarization exhibits distinct fatty acid metabolic dependencies: M1 polarization relies predominantly on glycolysis, whereas M2 polarization is coupled to elevated fatty acid oxidation (FAO), OXPHOS, and lipolysis. M2 macrophage FAO is driven by PPAR family members, particularly PPAR-γ and PPAR-δ, which promote OXPHOS to satisfy energy demands [127]. During *Mycobacterium tuberculosis* (Mtb) infection, pulmonary macrophages upregulate FAO through mechanisms partially dependent on TLR2-MAPK signaling and its downstream effector, dual-specificity phosphatase 5 (DUSP5). DUSP5 silencing elevates free fatty acid and triglyceride levels while suppressing FAO-related genes, including *CPT1A* and *PPAR-α*. This attenuates pro-inflammatory cytokine production (e.g., IL-1β, IL-6) and suppresses NF-κB signaling [132,133,134].

Tumor-associated macrophages (TAMs) exhibit functional dichotomy: M1-like TAMs enhance anti-tumor immunity through ADCC and antigen presentation, whereas M2-like TAMs promote tumor progression by inducing immune tolerance. Unsaturated long-chain fatty acids (LCFAs), such as oleic acid, drive M2 polarization through PPAR-γ signaling enhancement and FAO upregulation, thereby inducing immunosuppression. Tumor-microenvironment (TME)-derived protein S100A4 critically regulates TAM polarization through PPAR-γ and FAO modulation. S100A4-deficient macrophages fail to upregulate FAO, thereby maintaining pro-inflammatory and anti-tumor phenotypes [131].

FAO significantly influences DC differentiation and functionality. FAO and AMPK inhibition promote conventional DC differentiation, whereas diminished ROS enhances conventional DC1 proportions [135,136]. Beyond differentiation, FAO modulates DC functionality: Saturated fats activate TLRs to induce costimulatory molecules, MHC molecules, and pro-inflammatory cytokines, whereas polyunsaturated fatty acids (PUFAs) suppress LPS-driven pro-inflammatory signaling and prevent DC activation. TLR activation triggers de novo fatty acid synthesis and lipid droplet formation while downregulating FAO, thereby establishing an energy reservoir for DC activation and T-cell priming [136]. Nevertheless, excessive lipid accumulation impairs DC functionality and disrupts T-cell activation [137]. Furthermore, FAO is associated with tolerogenic DC development, which maintains immune tolerance through Treg induction and anti-inflammatory cytokine production (e.g., IL-10 and TGF-β) [137].

FAO is essential for neutrophil metabolism and functionality, particularly in glucose-deprived microenvironments where FAO generates energy through autophagy-mediated lipid catabolism [138]. In mature neutrophils, FAO is dispensable for basal energy production yet crucial for effector functions, including NETosis [139]. SCFAs additionally regulate neutrophil apoptosis, chemotaxis, and ROS production [140].

FAO additionally modulates lipid metabolic states and functionality of ILCs and NK cells. ILC2s, the predominant tissue-resident ILC population in pulmonary tissues, depend on FAO for pro-inflammatory [141]. In allergen-induced airway inflammation models, ILC2s internalize exogenous lipids (e.g., palmitic acid) and sequester them as lipid droplets. Subsequent lipid oxidation occurs through an IL-33-initiated, PPAR-γ- and diacylglycerol O-acyltransferase 1 (DGAT1)-mediated process, thereby expanding pro-inflammatory ILC2 populations within the lung [142]. In allergic asthma models, autophagy deficiency in activated ILC2s impairs FAO utilization, inducing a metabolic shift toward glycolysis. This disruption compromises ILC2 homeostasis and Th2 cytokine production while attenuating ILC2-mediated pulmonary inflammation [143]. During acute retroviral infection, NK cells upregulate CD36 expression, a critical mediator of FAs uptake. Activated NK cells exhibit metabolic flexibility, utilizing FAs (e.g., linoleic acid, palmitic acid, and oleic acid) as energy substrates, with elevated FAO correlating with enhanced cytotoxicity [144].

Metformin modulates fatty acid metabolism through diverse mechanisms. In intestinal epithelial cells, metformin suppresses apolipoproteins synthesis (ApoA-IV and ApoB-48) through AMPK and glucagon-like peptide-1 (GLP1) activation, thereby attenuating triglyceride synthesis and inhibiting chylomicron formation and secretion. Additionally, metformin diminishes intestinal bile acid reabsorption and enhances chylomicron clearance, consequently reducing circulating cholesterol. In adipose tissue and skeletal muscle, metformin promotes FAO, resulting in diminished adiposity, reduced very-low-density lipoprotein (VLDL-TG) levels, and decreased brown adipose tissue (BAT) lipid droplet content. Metformin enhances fatty acid utilization and oxidative phosphorylation through upregulation of mitochondrial respiratory chain components, activates lipolytic enzymes to stimulate lipolysis, and improves adipose tissue fatty acid uptake and utilization. Furthermore, metformin induces fibroblast growth factor 21 (FGF21) production, thereby reducing fat mass and enhancing FAO in white adipocytes of obese mice. In skeletal muscle, metformin attenuates lipid accumulation in ob/ob mice through FAO promotion and downregulation of genes involved in fatty acid synthesis and acyl-CoA metabolism [145].

Metformin modulates immune cell functionality and phenotypes through fatty acid metabolic regulation. In macrophages, metformin promotes M2 polarization through FAO and OXPHOS enhancement, thereby augmenting anti-inflammatory and tissue-reparative functions. In TAMs, metformin potentially suppresses FAO to inhibit M2 polarization, consequently enhancing anti-tumor activity [127]. Metformin disrupts lipid metabolic homeostasis in DCs, perturbing the balance between fatty acid synthesis (FAS) and FAO. Specifically, metformin enhances glucose utilization and glycolysis while suppressing the TCA cycle and pentose phosphate pathway (PPP) through forkhead box O3 (FoxO3) signaling. This induces fatty acid and lactate accumulation while diminishing DC synthetic metabolism, thereby generating metabolic states conducive to tolerogenic functionality [21]. Nevertheless, fatty acid metabolic mechanisms in other innate immune cell subsets require further investigation.

### 5.3. Epigenetic Reprogramming: Metformin Modulates Innate Immunity via DNA/Histone Modification and ncRNA Regulation

Epigenetics encompasses heritable alterations in gene activity without DNA sequence modification. Four primary mechanisms underlie epigenetic regulation: DNA methylation, chromatin structural modulation, histone post-translational modifications, and non-coding RNA (ncRNA)-mediated regulation [146]. These mechanisms profoundly modulate innate immune functions.

Metformin modulates DNA methyltransferases (DNMTs) and cellular metabolism via the AMPK signaling pathway, thereby remodeling the genome-wide DNA methylation landscape of immune cells. On the one hand, metformin activates AMPK to directly suppress the activity of DNMTs. On the other hand, it indirectly regulates the intracellular levels of DNMT inhibitors, triggering differential demethylation or hypermethylation in gene promoter regions and altering the expression of immune-related genes [147,148]. Clinical studies have demonstrated that short-term administration of metformin at therapeutic doses can alter the methylation status of 125 CpG sites in peripheral blood leukocytes from healthy individuals. Among these, protein O-fucosyltransferase 2 (POFUT2), calmodulin-dependent protein kinase kinase 1 (CAMKK1), and up-frameshift protein 1 (UPF1) are stably differentially methylated loci, which regulate genes involved in energy homeostasis and inflammatory responses and further mediate the functional changes in innate immunity [149]. In patients with type 2 diabetes, metformin intervention leads to methylation alterations at 26 CpG sites in peripheral blood, predominantly presenting as hypermethylation. Methylation changes at the PBX/Knotted homeobox 2 (PKNOX2), WD and tetratricopeptide repeat 1 (WDTC1), and MHC class I polypeptide-related sequence B (MICB) loci mediate the anti-inflammatory and hypoglycemic effects of metformin [150]. Mechanistically, metformin elevates the SAM:SAH ratio by regulating mitochondrial serine one-carbon metabolism and the activity of mitochondrial complex I, which promotes global DNA methylation. Meanwhile, it downregulates the expression of methylation- and demethylation-related genes such as DNA methyltransferase 1 (DNMT1) and Tet methylcytosine dioxygenase 1 (TET1), and it stabilizes the epigenetic status of immune cells [151,152].

Metformin differentially regulates the activity of various histone-modifying enzymes, mediating specific changes in histone acetylation, methylation, and lactylation and further modulating inflammation and functional activation of innate immune cells. In terms of histone acetylation regulation, metformin exerts divergent effects on histone acetyltransferase (HAT) family members: It increases the activity of HAT1 while inhibiting E1A-binding protein p300 (p300) and CREB-binding protein (CBP). It also indirectly suppresses class I/II histone deacetylases (HDACs) and enhances the activity of silent mating type information regulation 2 homolog 1 (SIRT1), a longevity-associated class III HDAC, to maintain cellular homeostasis and immune balance [153,154,155,156].

For histone methylation, metformin alters histone methylation patterns by regulating the expression of histone methyltransferases (HMTs). Its regulatory effects on immune gene expression are either activating or repressive depending on the cellular microenvironment [157]. Additionally, metformin directly binds to and inhibits methyltransferases, including coactivator-associated arginine methyltransferase 1 (CARM1) and protein arginine methyltransferase 6 (PRMT6); reduces the levels of histone H3 asymmetric dimethyl-arginine 17 (H3R17me2a) and histone H3 arginine 2 asymmetric dimethylation (H3R2me2a) modifications; and participates in the regulation of metabolism- and immune-related genes [158,159]. In inflammatory immune cells, metformin reverses the LPS-induced elevation of histone H3 lysine 14 (H3K14) acetylation in monocytes, inhibits the secretion of chemokines interferon γ-inducible protein 10 (IP-10) and monocyte chemoattractant protein-1 (MCP-1), and alleviates inflammatory responses [160,161]. In a zebrafish inflammation model, metformin downregulates H3K18 lactylation in neutrophils, reduces ROS production and neutrophil recruitment, and suppresses local inflammation [90]. Furthermore, metformin modulates the proliferation and apoptosis of immune cells by regulating H3K9 and H3K18 acetylation in combination with long non-coding RNA (lncRNA) regulatory pathways [162].

Metformin regulates the functions of innate immune cells at the post-transcriptional level by modulating the biogenesis and expression of non-coding RNAs. It upregulates the expression of endoribonuclease dicer (DICER), a key enzyme for microRNA (miRNA) biosynthesis, and thereby modulates the expression of multiple miRNAs associated with various diseases and immunity [163]. In natural killer (NK) cells, metformin upregulates immunostimulatory miR-150 and miR-155 and downregulates immunosuppressive miR-146a. These changes markedly enhance NK cell cytotoxicity, increase the expression of NKp46, FasL, and IFN-γ, inhibit the secretion of anti-inflammatory cytokine IL-10, and remodel the immune functions of NK cells [105]. Moreover, metformin regulates the expression of lncRNAs such as metastasis-associated lung adenocarcinoma transcript 1 (MALAT1), which is involved in inflammatory modulation and anti-tumor immunity. Its regulatory actions cover glucose metabolism and systemic inflammation across multiple tissues and cell types [162,164].

The epigenetic regulatory effects of metformin are tightly linked to its profound impacts on cellular metabolism. Accumulating evidence indicates that metformin activates AMPK to modulate the activities of epigenetic modifying enzymes, including HATs, HDACs, and DNMTs, consequently altering histone and DNA modifications as well as the expression of downstream genes [165]. Meanwhile, metabolite-derived chromatin modifications (e.g., acetyl-CoA, α-ketoglutarate, and SAM) are modulated by metformin at both local and systemic levels. By targeting global metabolic pathways or enzymes responsible for generating chromatin-modifying metabolites, metformin indirectly governs the activation state of chromatin remodelers. It also affects the interactions among metformin, diet, and gut microbiota, thereby systematically regulating metabolic inputs to chromatin [166]. This coupled metabolism–epigenetics mechanism provides a theoretical basis for the therapeutic potential of metformin in non-diabetic disorders such as cancer.

## 6. Future Perspectives and Clinical Translation: Decoupling Efficacy from Toxicity

Despite the increasingly recognized immunomodulatory potential of metformin, its clinical translation remains constrained by an unresolved tension between efficacy and toxicity. This dichotomy stems from its dual mechanism of action. On the one hand, mitochondrial complex I inhibition effectively suppresses tumorigenesis [167] and attenuates excessive inflammatory responses [168]. On the other hand, elevated systemic exposure to this same mechanism may precipitate global energetic deficit, thereby increasing lactic acidosis risk [169] and gastrointestinal intolerance. Conversely, sub-therapeutic dosing, while safer, may fail to achieve the threshold necessary for AMPK-dependent immunometabolic reprogramming. Thus, the central challenge lies not in establishing metformin’s efficacy but in optimizing its deployment to preserve therapeutic benefits while circumventing systemic metabolic stress.

To address this, future translational efforts should move beyond conventional dose escalation and instead focus on mechanism-informed precision strategies. ① Targeting Alternative Pathways to Reduce Complex I-Dependent Toxicity: Emerging evidence suggests that metformin engages additional molecular targets beyond mitochondrial complex I. Notably, PEN2, a component of the lysosomal v-ATPase–Ragulator complex, has been identified as a critical mediator of metformin-induced AMPK activation at the lysosomal surface [170]. In parallel, mitochondrial glycerol-3-phosphate dehydrogenase (mGPD) has also been implicated as a direct target influencing redox balance and gluconeogenesis [171]. These findings open the possibility of designing next-generation biguanides or small molecules that selectively engage the PEN2-AMPK axis without profound inhibition of mitochondrial respiration. Such an approach could, in principle, retain immunometabolic benefits while mitigating lactate accumulation and systemic toxicity. ② Tissue- or Cell-Specific Delivery Strategies: Another promising direction lies in improving the spatial precision of drug delivery. Nanoparticle-based systems have been explored to selectively deliver metformin to specific tissues, including lymphoid organs and the tumor microenvironment (TME), thereby achieving locally effective concentrations while minimizing systemic exposure [172]. Targeting immune niches such as tumor-associated macrophages (TAMs) or Treg may enhance immunomodulatory efficacy without amplifying off-target toxicity in metabolically sensitive organs such as the liver or intestine. This spatial decoupling of exposure and effect represents a critical step toward improving the therapeutic index. ③ Precision Medicine and Patient Stratification: Inter-individual variability in response to metformin is increasingly recognized as a major determinant of clinical outcome. Genetic polymorphisms in organic cation transporters (OCT1/SLC22A1), which regulate the cellular uptake of metformin, have been shown to influence both pharmacokinetics and therapeutic response [173]. In addition, baseline immunometabolic states may further modulate responsiveness. Future clinical trials should therefore incorporate molecular stratification and metabolic phenotyping to guide dosing strategies. Such an approach may allow maximization of immunological benefits within a defined safety window rather than relying on uniform dosing paradigms.

In summary, the future of metformin as an immunomodulatory agent does not lie in increasing dose intensity but in refining target engagement. Decoupling beneficial signaling pathways—such as AMPK/PEN2 activation—from the deleterious consequences of mitochondrial respiratory inhibition will be essential. Achieving this balance will likely determine whether metformin can be repositioned as a safe and effective therapy for cancer and inflammatory and metabolic diseases. Table 2 summarizes the potential applications of metformin.

## 7. Conclusions

Metformin is a multifunctional immunometabolic regulator with metabolic and immune-modulating properties. Its anti-inflammatory efficacy in type 2 diabetes is clinically validated, and preclinical and early clinical studies have revealed its promising potential in chronic inflammatory diseases, acute respiratory distress syndrome, and tumor immunotherapy. However, its roles in tissue repair, infection control, and trained immunity remain only theoretical, requiring further clinical verification.

Current mechanistic studies of metformin rely largely on animal and in vitro experiments that cannot fully simulate human pharmacological conditions. Existing clinical data are mainly derived from diabetic patients, rendering it difficult to define its immune-modulatory dosage independent of hypoglycemic effects. In addition, the long-term safety of its persistent remodeling of innate immunity remains unclear.

Future studies should conduct clinical trials targeting immune endpoints in non-diabetic populations and optimize experimental models with physiologically equivalent drug concentrations. In addition, novel metformin derivatives and delivery systems should be developed, targeted drug delivery should be advanced, and metformin’s therapeutic potential in inflammatory and immune-related diseases should be maximized.

## Figures and Tables

**Table 1:** Comparison of preclinical and clinical evidence for metformin’s immunomodulatory effects.

Immune Cell	Primary Functions	Regulatory Mechanisms	Research Models	Research Limitations	References
Macrophages	Regulate polarization to balance inflammation and tissue repair; remodel tumor immune microenvironment and modulate mesenchymal stem cells	Activate AMPK; reprogram metabolism, eliminate ROS, and inhibit NF-κB; regulate gut microbiota; involve AMPK/PGC-1α/PPARγ, TLR4/NF-κB, and AMPK-STAT3/JNK1 cascades	In vitro cell models, animal models	Context-dependent effects; supraphysiological doses widely used in vitro; most data from preclinical studies	[35,36,37,44,45,46,47,48,49,54,55,56,57]
DCs	Control maturation/activation to balance immunity and tolerance; suppress plasmacytoid DCs with heterogeneous effects on conventional DC subsets	AMPK-driven immunometabolic remodeling; inhibit mitochondrial complex I; modulate gut microbial metabolites	In vitro cell models, animal models, and clinical studies	Effects vary with context and DC subtypes; most research stays at the preclinical stage	[73,74,75,77,78,79]
Neutrophils	Alleviate excessive inflammation and tissue damage; block infiltration, proinflammatory cytokines, and NET formation; enhance efferocytosis and anti-tumor immunity	Target AMPK; reshape metabolism to correct abnormal glycolysis/oxidative metabolism; maintain intestinal microbiota homeostasis	In vitro cell models, animal models, and clinical observational studies on cancer patients	Context-dependent efficacy; evidence mainly derived from preclinical experiments	[87,93,94,95]
NK cells/ILCs	Balance immune surveillance and inflammatory homeostasis; improve NK cytotoxicity and intratumoral infiltration; preserve ILC1/ILC3 survival and optimize ILC functions	Activate AMPK to coordinate glycolysis and OXPHOS; modulate miRNAs and mTOR/pSTAT1	In vitro cell models, animal models, and clinical trials on cancer patients	Divergent responses among ILC subsets; incomplete mechanistic elucidation; dominated by preclinical evidence	[103,104,105,106,108,109,110]

**Table 2:** Potential clinical applications of metformin in innate immunity modulation.

Indication Category	Type of Evidence	References
Type 2 diabetes mellitus	Large-scale clinical cohorts and meta-analyses of randomized controlled trials (RCTs)	[65,66]
Adjuvant tumor therapy and cancer chemoprevention	Small-sample clinical trials and retrospective cohort studies	[62,65,96,111]
Metabolic syndrome and obesity-associated chronic low-grade inflammation	Clinical observational studies	[64]
Prevention of atherosclerosis and adverse cardiovascular events	Clinical observation in patients with diabetes complicated by atherosclerosis; in vivo rat models of myocardial infarction and atherosclerosis plus vascular cellular experiments	[33,52,53,63,89]
Non-alcoholic fatty liver disease (NAFLD)	In vivo experiments in mouse NAFLD models and in vitro cellular assays	[54,55,56,57]
Multiple solid malignancies (lung cancer, pancreatic cancer, melanoma, colorectal cancer)	Tumor-bearing mouse models, tumor cell assays, and immune cell experiments	[36,92,93,103,107,108]
Allergic asthma and airway hyperresponsiveness	Mouse models of allergic airway inflammation	[110]
Autoimmune diseases	In vivo animal models of autoimmune diseases combined with immune cell assays	[73,79,90]
Multiple organ injury and tissue repair (lung, nerve, bone, and cutaneous wound)	Injury models in neonatal mice and rats plus cellular experiments	[35,43,44,45,46,47,48,49,50,89,90,91]

## Data Availability

No new data were created or analyzed in this study.
